# Exploring the Impact of Alginate—PVA Ratio and the Addition of Bioactive Substances on the Performance of Hybrid Hydrogel Membranes as Potential Wound Dressings

**DOI:** 10.3390/gels9060476

**Published:** 2023-06-09

**Authors:** Diana Stan, Elena Codrici, Ana-Maria Enciu, Ewa Olewnik-Kruszkowska, Georgiana Gavril, Lavinia Liliana Ruta, Carmen Moldovan, Oana Brincoveanu, Lorena-Andreea Bocancia-Mateescu, Andreea-Cristina Mirica, Dana Stan, Cristiana Tanase

**Affiliations:** 1 DDS Diagnostic, 031427 Bucharest, Romania; pharmaco@ddsdiagnostic.com (L.L.R.); research@ddsdiagnostic.com (L.-A.B.-M.); research.imuno@ddsdiagnostic.com (A.-C.M.); dana_stan@ddsdiagnostic.com (D.S.); 2 Doctoral School of Medicine, Titu Maiorescu University, 040441 Bucharest, Romania; 3 Victor Babes National Institute of Pathology, 050096 Bucharest, Romania; elena.codrici@ivb.ro (E.C.); ana.enciu@ivb.ro (A.-M.E.); bioch@vbabes.ro (C.T.); 4 Department of Cell Biology and Histology, Carol Davila University of Medicine and Pharmacy, 050474 Bucharest, Romania; 5 Department of Physical Chemistry and Physicochemistry of Polymers, Faculty of Chemistry, Nicolaus Copernicus University, 87-100 Toruń, Poland; ewa.olewnik@umk.pl; 6 Department of Bioinformatics, National Institute of Research and Development for Biological Sciences, 060031 Bucharest, Romania; georgi.gavril@yahoo.com; 7 National Institute for R&D in Microtechnology, 077190 Bucharest, Romania; carmen.moldovan@imt.ro (C.M.); oana.brincoveanu@imt.ro (O.B.); 8 Research Institute of the University of Bucharest, 060102 Bucharest, Romania; 9 Department of Cell Biology and Clinical Biochemistry, Faculty of Medicine, Titu Maiorescu University, 040441 Bucharest, Romania

**Keywords:** biohybrid, hydrogel, wound, healing, dressing, bioactive, collagen, hyaluronan

## Abstract

Healthcare professionals face an ongoing challenge in managing both acute and chronic wounds, given the potential impact on patients’ quality of life and the limited availability of expensive treatment options. Hydrogel wound dressings offer a promising solution for effective wound care due to their affordability, ease of use, and ability to incorporate bioactive substances that enhance the wound healing process. Our study aimed to develop and evaluate hybrid hydrogel membranes enriched with bioactive components such as collagen and hyaluronic acid. We utilized both natural and synthetic polymers and employed a scalable, non-toxic, and environmentally friendly production process. We conducted extensive testing, including an in vitro assessment of moisture content, moisture uptake, swelling rate, gel fraction, biodegradation, water vapor transmission rate, protein denaturation, and protein adsorption. We evaluated the biocompatibility of the hydrogel membranes through cellular assays and performed instrumental tests using scanning electron microscopy and rheological analysis. Our findings demonstrate that the biohybrid hydrogel membranes exhibit cumulative properties with a favorable swelling ratio, optimal permeation properties, and good biocompatibility, all achieved with minimal concentrations of bioactive agents.

## 1. Introduction

With a growing population and increased life expectancy, it is crucial for the healthcare system to adapt to the complex medical needs of patients, while the industry must provide accessible and cost-effective solutions. Recent research conducted in 2021 confirmed the significant impact of chronic wounds on patients’ quality of life [1]. Additionally, an analysis of Medicare data highlighted that approximately 2.5% of the U.S. population suffers from chronic wounds [2]. As the global population ages, diseases that can complicate with chronic wounds, such as diabetes or venous insufficiency, become more prevalent. Consequently, there is an increasing demand for wound management resources that are affordable, user-friendly, and effective. Despite significant advancements in wound management resources, including the highly sought-after “smart” dressings, there remains a significant gap between research findings and the availability of commercial products. This gap arises from several factors, such as the limited scalability of prototypes, the substantial costs associated with mass production or restricted access to innovative technologies in underdeveloped regions. Additionally, the high expenses related to regulatory affairs pose significant barriers to the entry of new products into the market [3]. In order to develop an effective and customized treatment plan, healthcare professionals need to integrate information about the patient, specific wound characteristics, and the properties of the dressing being considered. This process often demands significant time and resources, leading healthcare workers to often rely on traditional treatment strategies [4,5]. In the 1960s, the modern world rediscovered through the work of Winter [6,7], Hinman and Maibach [8] that wounds tend to heal better in a moist environment and the use of bandages gained widespread popularity. Since then, significant progress in the fields of material science, biotechnology, and medicine have led to notable advancements in the design and therapeutic efficacy of wound dressings. Today there are several materials that are popular among those used in wound dressing production such as natural and synthetic polymers. Natural polymers (biopolymers) such as proteins or polysaccharides have the great advantage of high biocompatibility, biodegradability and bioactivity although they also possess some disadvantages such as low mechanical strength, batch-to-batch variations or the need for further purification. Synthetic polymers offer advantages such as improved stability, higher reproducibility, and customizable properties, but they may also have disadvantages including poor biocompatibility and limited bioactivity [9,10,11,12]. Alginate (Alg), a highly versatile anionic hydrophilic natural polysaccharide primarily obtained from brown algae (phylum *Ochrophyta*, class *Phaeophyceae*), is composed of repeating mannuronic acid (M) and glucuronic acid (G) units [13,14,15]. Alginate extract is found as a sodium or calcium salt and contains numerous hydroxyl and carboxyl functional groups, enabling the formation of intramolecular hydrogen bonds [14,16,17,18,19]. Depending on the processing method, it can be produced as foams [20,21], sponges [22,23], hydrogels [24,25,26], films [27,28,29], nanofibers [30,31,32], or nanoparticles [33,34,35]. Alginate can be crosslinked via physical or chemical methods [14]. It can form hydrogels through ionic crosslinking or processing in an acidic environment with the formation of hydrogen bonds between its chains [26,36,37]. Alginate is biocompatible and biodegradable, non-toxic and non-irritant substance, has immunomodulatory and hemostatic effects and, thanks to its functional groups, has an impressive ability to absorb liquids [38,39]. During the processing stage, alginate can be combined with other natural or synthetic polymers such as hyaluronic acid [40], gelatin [41], or polyvinyl alcohol [41,42]. Polyvinyl alcohol (PVA), a hydrophilic synthetic polymer, is traditionally derived from the hydrolysis of polyvinyl acetate and is characterized by an abundance of hydroxyl groups that facilitate the formation of hydrogen bonds [43]. Products made from polyvinyl alcohol can be in the form of hydrogels [44], nanofibers, foams [45] or hydrocolloids [46]. Chemical crosslinking methods of polyvinyl alcohol use irradiation, radical polymerization, or crosslinking agents while physical methods use freeze–thaw cycles, irradiation or electrospinning [44,47,48,49,50,51]. PVA can be tailored or combined with other natural and/or synthetic polymers which influence the stiffness [52], biocompatibility [53] or microstructure [54] of the final product. Polyvinyl alcohol dressings are known for their transparency, semipermeability, strong mechanical resistance, and moisture retention abilities [43,44,55,56]. Collagen (COL) is a protein formed from amino acids such as hydroxyproline, glycine or proline that form polypeptide chains. Collagen fibers can be crosslinked via physical, chemical or biological methods [57] obtaining products in the form of sponges, fibers, films or nanofibers. Collagen is usually combined with other types of natural or synthetic polymers [58,59,60,61,62]. Collagen is naturally found at the tissue level in the extracellular space and gives mechanical resistance and elasticity to the skin while its fibers allow cell recruitment, attachment, proliferation and migration. When incorporated in wound dressings, collagen can promote the healing process [63,64,65,66]. Hyaluronic acid (HA) is a glycosaminoglycan consisting of repeating disaccharide units of β-D-glucuronic acid and N-acetyl-D-glucosamine, linked by glycosidic bonds, and can be obtained from animal or microbial sources [67,68,69]. The highly hydrophilic character is due to the abundant hydroxyl and carboxyl groups in its structure [69]. Hyaluronic acid dressings are available in various formats, including creams, sponges, films, hydrogels, and nanofibers. Free radical reactions, esterification, casting or electrospinning are some of the methods used to produce dressings that contain hyaluronic acid [70,71,72,73,74,75,76]. Hyaluronic acid is an essential part of the extracellular space, along with collagen. Thus, it is biocompatible, biodegradable and can be easily modified due to the presence of hydroxyl and carboxyl groups [71,73]. During the healing process, hyaluronic acid plays a role in various important aspects such as hemostasis, cell migration and proliferation, collagen deposition, angiogenesis, and re-epithelialization. It maintains a moist, optimal healing environment and has a supporting role for the cells involved in healing. The disadvantages of this biopolymer are its low mechanical strength and low stability, with rapid degradation [71]. 

An ideal wound dressing should incorporate a range of qualities, including the promotion of healing, biocompatibility, biodegradability, suitable mechanical properties, adaptability, moisture variations, semipermeability, conformity to body contours, ease of use, and affordability [77]. Due to the absence of a single substance possessing all the necessary characteristics for an ideal wound dressing, a combination of multiple substances may hold the key to developing highly performing dressings [10,78]. The formulation development and substance selection are dependent on specific requirements and desired properties. Our main goal is to explore the properties of a hybrid hydrogel membrane matrix made of PVA and alginate obtained through a scalable, non-toxic, eco-friendly method that incorporates bioactive substances such as collagen and hyaluronic acid for wound dressing applications in order to discover the most suitable formulation.

## 2. Results and Discussion

### 2.1. Formulation Optimization of Hydrogel Membranes

**Figure 1:**
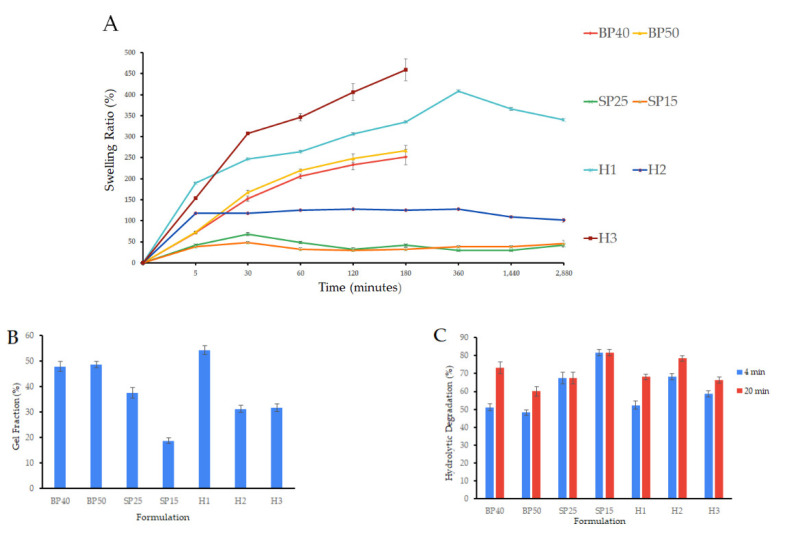
Initial evaluation of hydrogel formulations showing cumulative properties of hybrid hydrogel membranes, (**A**) swelling index, (**B**) gel fraction, and (**C**) hydrolytic degradation (dH_2_O, pH 7.4). Swelling index assay displays high SD due to variable disintegration rates of hydrogel samples during the study. Results are presented as mean ± S.D.

To ascertain the most performant formulation that could act as a matrix for integrating bioactive additives, we conducted an initial evaluation of seven formulations using alginate (Alg) and polyvinyl alcohol (PVA). We evaluated hydrogel membranes made of biopolymer (BP40 and BP50), synthetic polymer (SP25 and SP15), or a combination of both (hybrid hydrogel membranes—H1, H2and H3) macroscopically and via in vitro studies for their swelling index, degradation rate and gel fraction. As presented in Figure 1A, biopolymer hydrogel membranes showed a high swelling ratio (266.44 ± 12.82% at 3 h for sample BP50), however, the formed network was quickly destructured. In contrast, synthetic hydrogel membranes showed great stability at 48 h yet their swelling ratio did not exceed 67.88 ± 3.67%, especially for sample SP25 at 30 min. Remarkably, formulations BP40 and BP50 appeared to perform slightly better with higher gel fractions (Figure 1B) and lower degradation rates (Figure 1C) compared to SP25 and SP15. This unexpected observation contradicts the prevailing trend in the literature, which generally postulates that hydrogel membranes based on synthetic polymers perform superiorly to those based on biopolymers [79,80]. We presume that factors such as polymer molecular weight and concentration, the inclusion of glycerol, or the crosslinking method might account for the unique behavior of the hydrogel membranes obtained in this study. As depicted in Figure 1, the hybrid hydrogel membranes demonstrated cumulative properties consistent with those in previous literature reports [81,82]. Notably, formulation H1 demonstrated excellent swelling abilities, the highest gel fraction value and an acceptable degradation rate. These results are potentially attributed to the capability of Alg to crosslink through dual mechanisms: ionic interactions and freeze–thaw cycles [83,84]. This dual crosslinking approach may have facilitated the formation of a greater number of crosslinking sites in the hybrid hydrogel membrane, resulting in a denser network with a higher gel fraction and increased stability [80]. Interestingly, most researchers observe a decline in the gel fraction when combining biopolymers at increasing proportions with synthetic polymers. [79,85]. Consistently with the earlier results of this study, formulation H3 showed notable swelling ability but was quickly destructured after only 3 h. Similarly, sample H2 showed high stability but a diminished swelling capacity, illustrating the influence of the polymer ratios on the properties of the hydrogels.

Table 1 provides information on the pH, thickness, weight variations, and visual appearance of the hydrogel membranes. All formulations produced membranes that displayed some degree of skin adhesion and a cooling, pleasant feeling when placed on intact skin. While the synthetic polymer membranes exhibited distinct imprint patterns of crystalline structures, likely a result of freeze–thaw treatment, the incorporation of Alg eliminated this appearance in the case of hybrid hydrogel membranes. The hybrid membranes had excellent visual appearance but lost some degree of transparency. They were easy to manipulate and showed the best properties for the evaluated parameters, with cumulative properties. Based on these initial results, we selected formulations H1 and H2 for further experimental design, which involved the incorporation of bioactive compounds and an evaluation of various relevant parameters.

**Figure 2:**
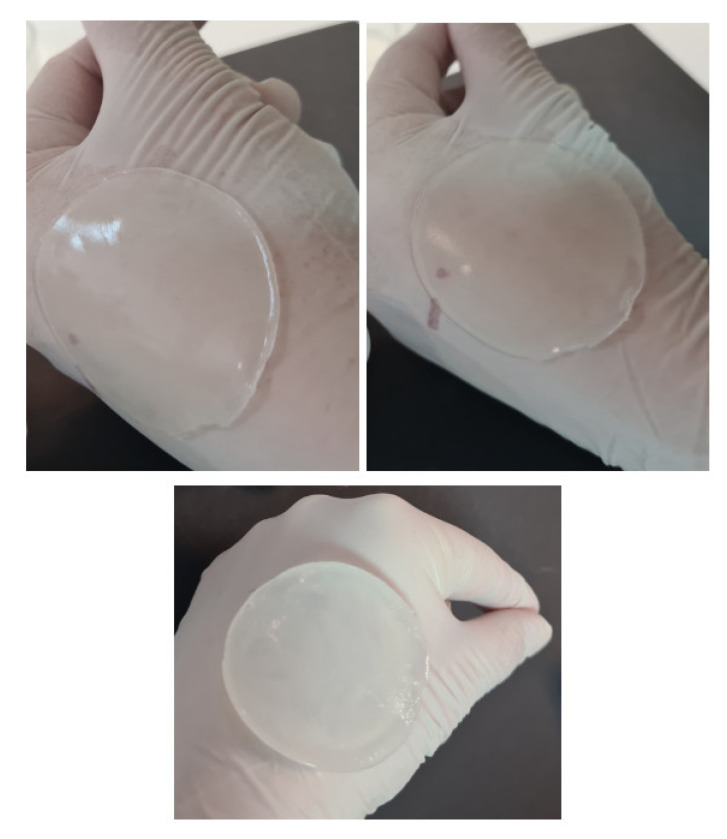
Visual representation of different biohybrid hydrogel membranes (BH). Note the transparency.

The incorporation of different concentrations of collagen (COL) and hyaluronic acid (HA) had no observable effect on the appearance of the biohybrid hydrogel membranes (Table 1). However, all optimized formulations required a longer time for solvent evaporation compared to the initial formulations and appeared thicker and juicier. Formulation BH2.80 had a high degree of hydration and a less stable structure, requiring gentle manipulation. The pH values for the biohybrid hydrogel membranes were in the neutral spectrum, ranging from 7.1 ± 0.01 to 7.28 ± 0.02. While most authors consider that a slightly acidic environment is more suitable for healing certain types of wounds [86,87,88], a recent study suggested that an alkaline environment could be more beneficial [89]. Therefore, the pH of the biohybrid hydrogel membranes obtained in this study is considered suitable for wound healing applications. Representative images of biohybrid hydrogel membranes enhanced with different concentrations of bioactive compounds—collagen and hyaluronic acid—are shown in Figure 2.

### 2.2. In Vitro Evaluation

#### 2.2.1. Moisture Content and Moisture Uptake

**Figure 3:**
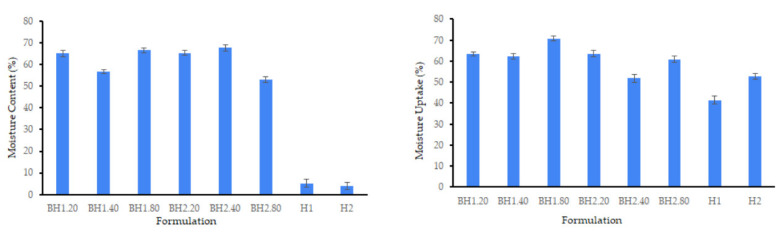
Moisture content and moisture uptake for biohybrid hydrogel membranes (BH) compared to hybrid hydrogel membranes (H). Results are presented as mean ± S.D.

As depicted in Figure 3, the incorporation of bioactive substances, specifically COL and HA, led to a substantial increase in the moisture content of the samples. The moisture content of sample H1 increased from 5.17 ± 1.89% to a maximum of 66.53 ± 0.95% for sample BH1.80, while for sample H2 it increased from 3.98 ± 1.51% to a maximum of 67.79 ± 1.45% for sample BH2.40. This significant increase in hydration can be attributed to the hydrophilic nature of collagen and hyaluronic acid, which have the ability to absorb and retain water molecules [67,68,90,91,92,93,94]. The moisture content of the samples was not significantly affected by changes in hyaluronic acid concentration, indicating that the lowest concentration tested may be sufficient for optimal outcomes. The observed increase in moisture content is of particular significance in biomedical applications, where it can influence the materials’ properties, biocompatibility, and ability to support cellular growth and tissue regeneration [95,96].

As presented in Figure 3, all samples had excellent moisture uptake capabilities achieving satisfactory levels of hydration, after undergoing the drying process. Notably, formulations BH1.40 and BH1.80 exhibited slightly higher values for moisture uptake compared to those for moisture content, demonstrating their excellent ability to attract water and maintain moisture. These results emphasize the great potential of the biohybrid hydrogel membranes obtained in our study for diverse biomedical applications, particularly in the realm of wound healing [96,97,98]. The moisture uptake ability of hydrogel membranes used for wound healing applications is an essential parameter, as it is related to their capacity to absorb wound exudate. 

#### 2.2.2. Swelling Ratio

**Figure 4:**
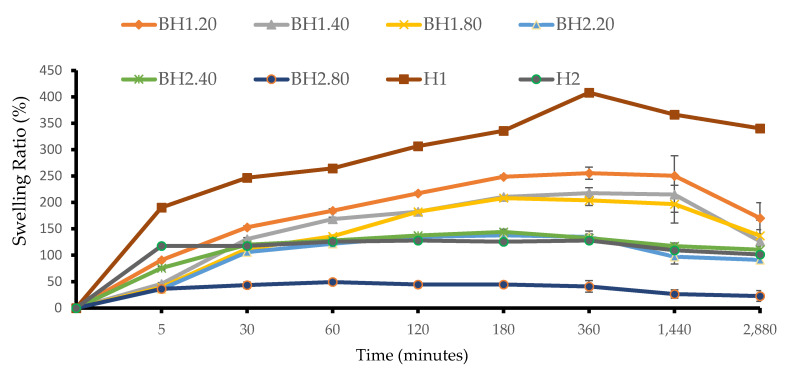
Swelling ratio of biohybrid hydrogel membranes (BH) to that of hybrid hydrogel membranes (H). Results are presented as mean ± S.D.

The swelling ratio, which indicates the water retention capacity of the hydrogels, was determined via a gravimetric assay. In order to mimic in vivo conditions, the samples were tested immediately following the completion of the crosslinking procedure and not fully dried prior to the analysis. The addition of COL and HA affected the swelling behavior differently depending on the PVA/Alg ratio, as depicted in Figure 4. Among the BH1 formulations, the sample with the lowest concentration of hyaluronic acid, BH1.20, exhibited the highest swelling ratio with a maximum value at 6 h. When compared to formulation H1, which also had the highest swelling ratio at 6 h (407.96 ± 2.34%), formulation BH1.20 had a lower value (255.44 ± 11.54%), and this was expected as BH1 formulations had a higher moisture content. Formulation BH1.80 had a maximum swelling rate at 3 h with a value of 208.40 ± 2.38%. The BH2.40 formulation exhibited a maximum value of 144.25 ± 4.98% at 3 h, which was slightly higher than the value of 125.58 ± 1.30% observed for sample H2. The increase was observed consistently from 30 min until the end of the 48 h assay. The swelling ratio of formulation BH2.80 was the lowest among that of all formulations, never exceeding 50%. These results align with previously reported data [67,99]. We assume that our findings are due to the way the bioactive substances interacted with the polymer chains before crosslinking, but also their influence on the behavior of the network formed after the crosslinking process was finished. COL and HA could have interposed between the polymer chains, affecting both the quality and the density of the bonds during the crosslinking process [100,101]. Following the crosslinking, the presence of highly hydrophilic bioactive compounds may exert additional forces on the resulting network by attracting and retaining additional amounts of water [67,93]. The non-toxic, environmentally friendly dual crosslinking method utilized in this study lead to the formation of non-covalent bonds and as a consequence the resulting network was more fragile. These assumptions are supported by analyzing the swelling behavior of the biohybrid hydrogel membranes compared to that of the hybrid hydrogel membranes. Interestingly, the optimal swelling index was observed in the case of formulation H1, both with and without the bioactive substances. This finding suggests that these formulations may have the ideal composition for producing high-performance biohybrid hydrogel membranes and that even discrete concentrations of bioactive substances may influence hydrogel properties [102,103]. 

#### 2.2.3. Biodegradation Study

**Figure 5:**
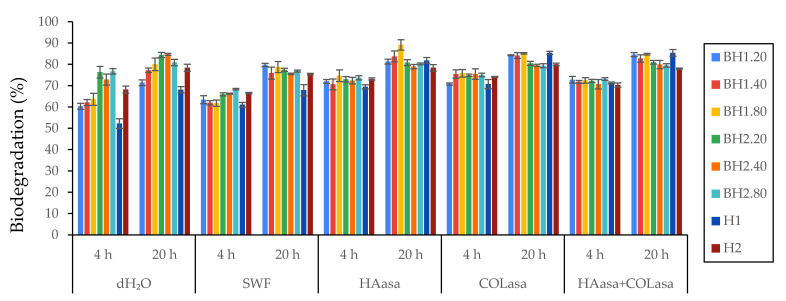
In vitro biodegradation study for biohybrid hydrogel membranes (BH) compared to hybrid hydrogel membranes (H) using distilled water (dH_2_O, pH 7.4), SWF (pH 8.3), HAasa (10 U/mL), COLasa (10 U/mL) and mixture of HAasa + COLasa (10 U/mL each). Results are presented as mean ± S.D.

The biodegradation of hydrogel membranes primarily occurs through the action of enzymes that recognize and target specific regions within the polymer network formed through crosslinking [104,105,106]. These enzymes facilitate the cleavage of bonds between polymer chains, resulting in the structural breakdown of the network. Physiologically, wound sites are abundant with enzymes such as collagenases, gelatinases, serine proteases, and glycosidases which can accelerate the degradation of topically applied products. In an in vivo setting, the enzymatic degradation rate of hydrogel membranes is influenced by various factors, including enzyme concentration, exposure time, pH, temperature, composition, and the material properties. In order to understand the degradation behavior of the biohybrid hydrogel membranes under circumstances that resemble in vivo conditions, we performed a degradation assay with different aqueous solutions that contained enzymes such as hyaluronidase (HAasa, 10 U/mL), collagenase (COLasa, 10 U/mL), and a mixture of the two (HAasa + COLasa, 10 U/mL each), compared to that with distilled water (dH_2_O, pH 7.4) and simulated wound fluid (SWF, pH 8.3). As expected, the addition of enzymes resulted in an elevated degradation rate of the samples, although the increase was not substantial but rather discrete. The results of the degradation study illustrated in Figure 5 demonstrate that the hydrogel membranes exhibit a non-specific degradation behavior, likely due to the hydrophilic nature of the formulations and the crosslinking method used, which allows water molecules to easily penetrate the structure. The rate of degradation primarily depends on the physical and chemical properties of the hydrogel, such as its hydrophilicity, crosslink density, and porosity, rather than its interaction with specific biological molecules. These findings suggest that the formulations may be suitable for incorporating and releasing therapeutic agents in a controlled manner that is not influenced by environmental factors, especially where quick delivery is required [107]. Additionally, it indicates that if the hydrogel membrane is employed as a wound dressing, it can integrate effectively with the surrounding tissue and be comfortably removed without causing harm to the newly formed tissue [108,109]. However, it also implies that the structural integrity of the hydrogel is relatively weak and that it could require frequent replacement, potentially limiting its suitability for certain types of wounds or patients. According to the available literature, the components used in the production of hydrogel membranes have been found to be non-toxic and biocompatible. As a result, it is expected that the degradation products would also exhibit non-toxic properties, based on the available data [66,110,111,112,113,114,115].

#### 2.2.4. Gel Fraction

**Figure 6:**
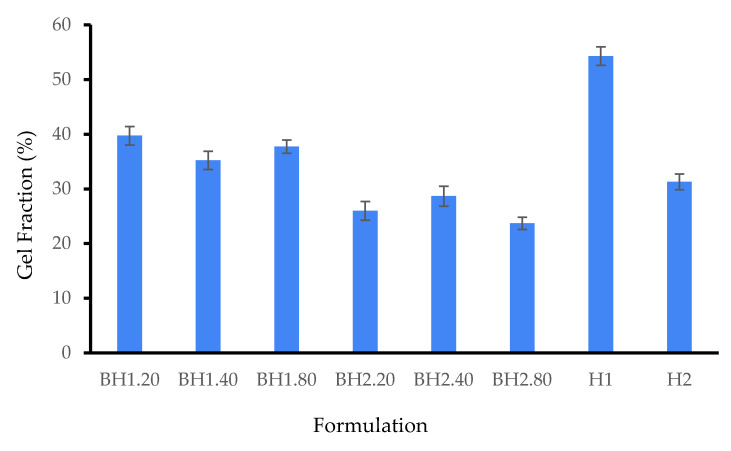
Gel fraction of biohybrid hydrogel membranes (BH) compared to that of hybrid hydrogel membranes (H). Results are presented as mean ± S.D.

The number of crosslinked molecules forming an insoluble gel fraction is reflected by the gel content of a hydrogel, while the non-crosslinked portion of the hydrogel dissolves upon immersion in a solvent, leading to a reduction in the sample weight [116]. In addition to PVA crosslinking, freeze–thaw cycles have been demonstrated to promote the formation of intermolecular bonds in Alg [84,117,118], collagen [101,119] and hyaluronic acid [100,120]. The biohybrid hydrogel membranes were found to exhibit lower values for the gel fraction when compared to the corresponding hybrid hydrogel membranes, as expected from previous assays. The gel fraction was in the range of 39.75 ± 1.69–23.72 ± 1.11% for samples BH1 and BH2 indicating low crosslinking. The results show that the variation of HA concentration had no influence on the gel content of the analyzed samples, and we assume that COL may have been mainly responsible for this effect. As depicted in Figure 6, BH1 samples have a slightly increased gel fraction. However, among all the biohybrid hydrogel membranes, formulation BH1.20 has the highest value, of 39.75 ± 1.69%. 

#### 2.2.5. Water Vapor Transmission Rate

**Figure 7:**
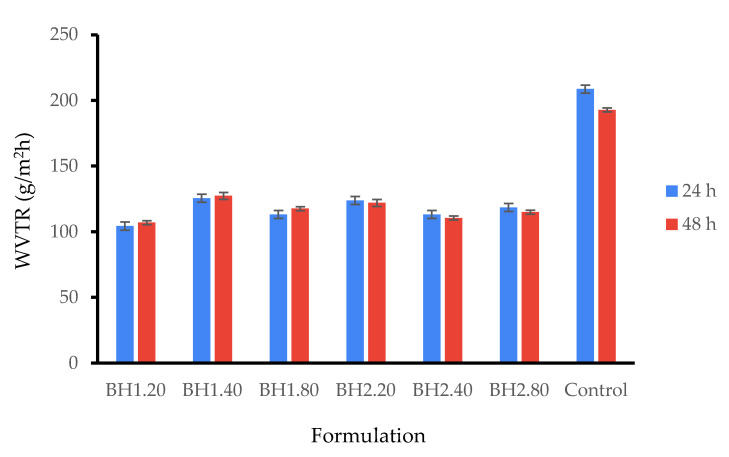
Water vapor transmission rate of biohybrid hydrogel membranes at 24 and 48 h. Results are presented as mean ± S.D.

In this study, we evaluated the water vapor transmission rate (WVTR) at 24 and 48 h using the gravimetric method while filter paper served as a control. At 24 h, the WVTR for the biohybrid hydrogel membranes was in the range of 104.33 ± 3.06–125.56 ± 3.06 g/m^2^ h and at 48 h between 106.99 ± 1.53–127.32 ± 2.65 g/m^2^ h. As it is shown in Figure 7, the WVTR of all formulations remained almost identical at 48 h, so we presume the biohybrid hydrogel membranes were stable and maintained their properties throughout the study. While these results align with those in the previous literature, it is important to note that there is no gold standard for WVTR and a wide range of values have been reported in the literature [82,96,121,122,123]. Water vapor transmission rate (WVTR) is a critical parameter for materials used in biomedical applications, particularly those intended to support the dermo-epidermal healing process that need to display semipermeability properties. This parameter indicates the ability of biohybrid hydrogel membranes containing bioactive compounds to facilitate gas exchange between the wound bed and the atmosphere while serving as a barrier against the infiltration of pathogens and preventing dehydration [124]. It is noteworthy that for intact and healthy skin, WVTR is typically in the range of 200–300 g/m^2^ h, while for wounded skin it can increase by a factor of ten or more, depending on the wound thickness [125,126]. 

#### 2.2.6. Protein Adsorption Study

**Figure 8:**
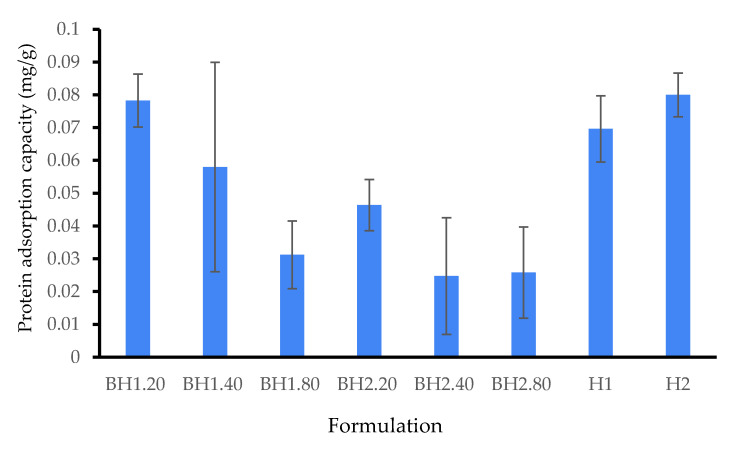
Protein adsorption study at 24 h. The amount of BSA that remained after removing the hydrogels was assayed spectrophotometrically using the Bradford method. The protein uptake was evaluated indirectly, taking into account the initial and equilibrium concentrations of BSA in the solution, as well as dimensions as the samples. Results are presented as mean ± S.D.

In order to evaluate the protein adsorption of the biohybrid hydrogel membranes, we implemented a modified protocol inspired by one in the existing literature [79,127]. Bovine serum albumin (BSA) was utilized as a model protein for this purpose, as it is known for its versatility and presence in wound exudate [128,129,130]. Our experimental findings (Figure 8) showed that the biohybrid hydrogel surface exhibited minimal protein uptake. Interestingly, the results demonstrated that protein uptake was particularly low in HA hydrogels. We presume that this finding can be attributed to the electrostatic repulsion forces between BSA and the negatively charged HA, in pH 7 buffer solutions. The experimental data also indicated a modest correlation between the concentration of HA and BSA uptake. It is well-known that a material used as a wound dressing that displays low protein adsorption has significant implications such as reduced inflammation at the wound site. The low protein adsorption promotes a moist wound environment, facilitating better interaction between the wound bed and the dressing, thereby enhancing wound healing. Additionally, the dressing requires fewer changes, minimizing disruption to the wound bed and improving patient comfort. The low protein adsorption of hydrogel membranes also helps prevent the accumulation of proteins that can promote bacterial growth, reducing the risk of secondary infections. Furthermore, it enhances patient comfort by reducing dressing adherence and pain during removal. [131,132,133]. 

#### 2.2.7. Protein Denaturation Study

**Figure 9:**
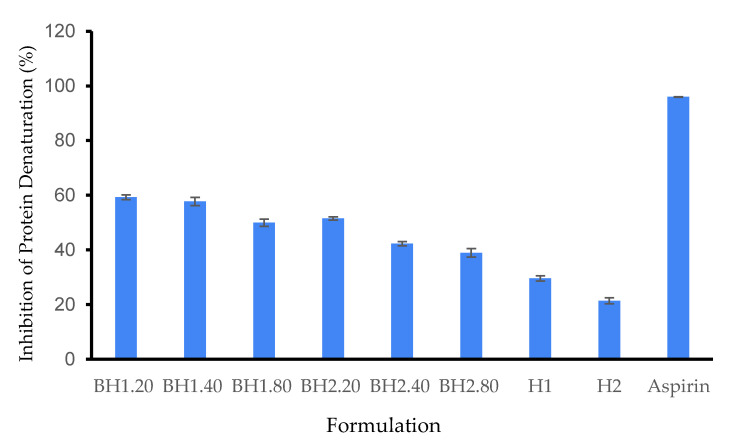
Inhibition of protein denaturation. Different hydrogel formulations were incubated in a BSA 5% solution at 37 °C, 15 min, then 70 °C for 70 min and cooled down to 25 °C. The samples were removed and the absorbance of the remaining BSA solution was spectrophotometrically assayed at 278 nm, using an aspirin solution (0.5 mg/mL) as a control. Results are presented as mean ± S.D.

The hydrogels were assessed for their anti-inflammatory properties using the protein denaturation inhibition assay, using BSA as a model protein and aspirin as a control. According to our results, the BH1.20 formulation exhibited the most significant inhibition of protein denaturation, with a value of 59.26 ± 0.86% in comparison to aspirin. We presume that this effect is primarily attributed to the presence of COL and HA in the formulation (Figure 9). Additionally, we observed that the inhibitory action had a tendency to be dose-dependent for hyaluronic acid, although higher concentrations led to a slight attenuation of the positive effect. This attenuation could be due to electrostatic repulsion forces between the negatively charged BSA and hyaluronic acid at pH 7. Comparing the BH1 and BH2 formulations, BH1 exhibited a superior protein inhibition rate, which can be correlated with the higher concentration of Alg in its hydrogel matrix. The methodology of this assay was adapted from the recent literature [134,135,136]. BSA is known to absorb strongly in the UV region due to its aromatic amino acids, such as tryptophan and tyrosine. Its UV-Vis spectrum exhibits two absorbance peaks at 280 nm (due to tryptophan absorption) and 220 nm (due to other aromatic amino acids) [137]. The specific shape and intensity of the spectrum can differ based on various factors, including the concentration of the protein, the pH level of the solution, and the existence of other molecules that interact with the protein. We evaluated the activity of BSA in PBS (pH = 7) on the entire spectrum, and the most significant variations appeared in the UV-Vis spectrum at 280 nm, so our further determinations were conducted at 280 nm.

### 2.3. LDH Assay

**Figure 10:**
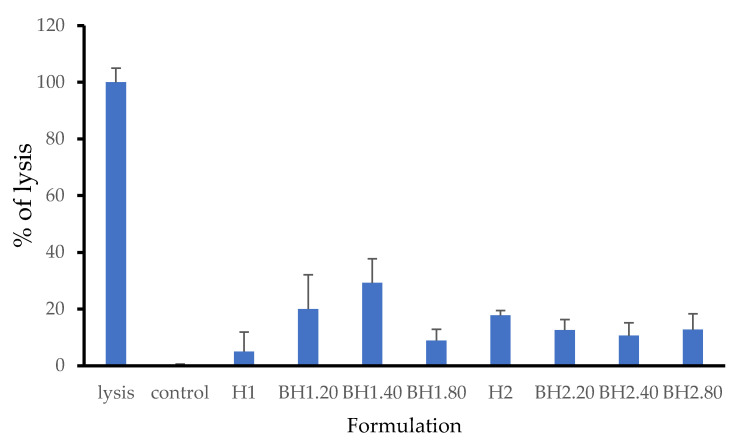
Cytotoxicity determined via LDH assay. Normal human fibroblasts were incubated with mentioned gel formulations. LDH release was measured spectrophotometrically in the cell supernatant. Readings were normalized to background for each formulation and expressed as ratio to normalized lysis control. Results are presented as mean ± S.D.

The assessment of in vitro cytotoxicity is crucial in determining the biocompatibility of biomaterials, and one commonly used method is the LDH assay (lactate dehydrogenase assay). LDH is a stable enzyme present in the cytosol of cells, and its release into the surrounding medium indicates cellular membrane permeabilization induced by chemicals or factors that affect cellular integrity [138,139]. In our study, the results obtained from the LDH assay revealed that the presence of collagen and hyaluronic acid had distinct effects on the response of human fibroblasts to the biohybrid hydrogel membranes (Figure 10). Specifically, cells treated with sample H1 showed a lower release of LDH compared to cells treated with sample H2. The introduction of bioactive compounds resulted in a decrease in LDH release for samples H2.20, H2.40, and H2.80 compared to sample H2. Surprisingly, the introduction of bioactive agents led to an increase in LDH release for cells treated with samples H1.20, H1.40, and H1.80. The highest LDH value recorded was for sample H1.40, reaching 29.24%. Notably, despite sample H1 demonstrating better overall performance compared to sample H2, the addition of COL and HA had a contrasting effect. Of all the tested formulations, H1 and BH1.80 showed the best results.

Several factors may account for these findings, including potential variations in the purity or molecular weight of the natural components employed in the study. Additionally, physical interactions due to the surface morphology and mechanical properties of the hydrogels could play a role, along with possible concentration-dependent effects. The specialized literature reported minimal cytotoxicity for the components used in the study. Charron et al. (2020) demonstrated that the number of freeze–thaw cycles (F-H cycles) did not have an influence on the cytotoxicity of gels incorporating polyvinyl alcohol (PVA) and gelatin [140]. Schulze et al. (2016) reported that the addition of PVA lowered the cytotoxicity of the hydrogel, due to its ability to incorporate and control nanoparticle release [141] while Song et al. (2012) demonstrated that adding HA to a PVA matrix positively influenced cellular growth, whereas adding collagen had minimal impact [142]. Another study demonstrated that collagen had no cytotoxic effect on human dermal fibroblasts for both non-crosslinked and crosslinked samples of hydrogel [143]. Interestingly, one study showed that an increase in collagen concentration (from 1% to 10%) leads to a slight increase in cytotoxicity levels [144]. In a study conducted by Travan et al. (2016) using human dermal fibroblasts, it was observed that there was no statistically significant difference in LDH release between untreated cells and cells treated with samples containing alginate and hyaluronic acid [145]. Another study showed that the addition of high concentrations of a hydrogel based on methylcellulose and hyaluronic acid lead to increased LDH release (up to 43%) [146]. Rubert et al. (2012) evaluated the cytotoxicity of alginate and hyaluronic acid hydrogels on MC3T3-E1 cells. The study found no toxicity in any of the tested hydrogels. Notably, comparing 1% hyaluronic acid hydrogels to 1% alginate hydrogels revealed a significant increase in cell viability [147]. Another study showed that samples containing gelatin and hyaluronic acid showed a greater effect on cell viability compared to samples containing collagen alone on mouse fibroblasts [148] while other authors reported that the addition of HA has a positive effect on cell viability and causes a decrease in LDH release [149,150].

### 2.4. Instrumental Evaluation

#### 2.4.1. Morphological Evaluation

**Figure 11:**
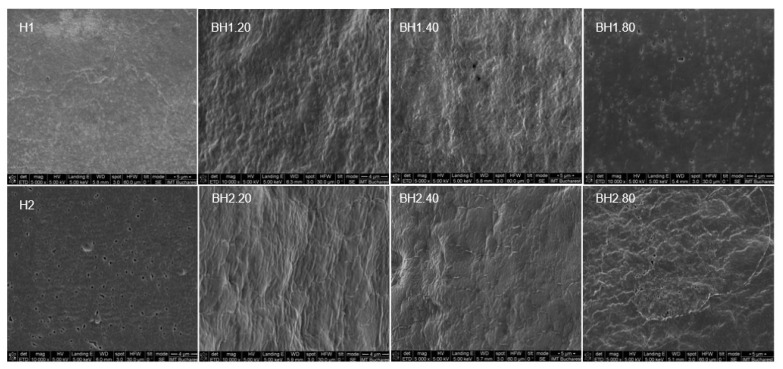
Surface morphology of hybrid hydrogel membranes (H) and biohybrid hydrogel membranes (BH) after 48 h of drying at 55 °C.

**Figure 12:**
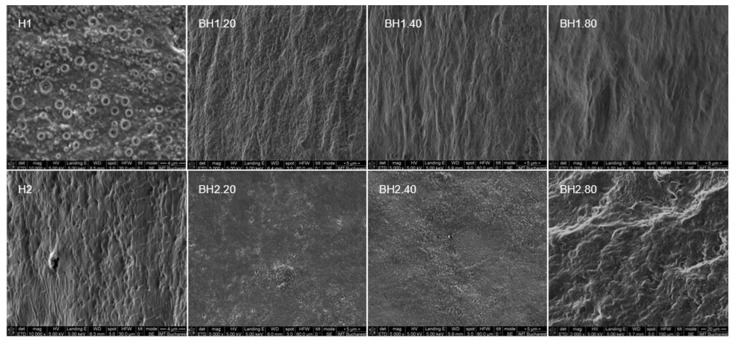
Morphology of cross-section of hybrid hydrogel membranes (H) and biohybrid hydrogel membranes (BH) after 48 h of drying at 55 °C.

The scanning electron microscope (SEM) images (Figure 11 and Figure 12) revealed that hybrid hydrogel membranes exhibit a predominantly smooth surface, interspersed with pores of varying dimensions that can also be observed on the cross-section. These pores are relatively uniformly distributed across both the surface and the cross-section of the membranes. On closer inspection, there were minimal presence of aggregates or crystals, and negligible ripples, lumps, or gaps on the surface of the hydrogels. This suggests a homogeneous blending of the two constituent polymers. Compared to sample H2, sample H1 presented a less porous surface. Upon the incorporation of bioactive substances—COL and HA—an increase in surface roughness was observed for the biohybrid hydrogel membranes. This change could potentially be attributed to the infiltration of these bioactive substances into the free spaces of the established network as suggested by previous assays. Interestingly, the alterations in pore size and distribution were less noticeable in the hydrogel samples containing the highest concentration of HA. For sample H1.80, the SEM images revealed an increase in the number of pores, implying a more pronounced effect of the variance of HA concentration on this particular hydrogel formulation. While the variation in the PVA/Alg ratio for the formulations investigated in this study did not significantly affect the surface morphology, pore size, or distribution, the general appearance of the hydrogel membranes was nonetheless consistent with that in the previously published literature [93,151,152]. This could indicate that factors other than the PVA/Alg ratio might influence membrane characteristics such as the addition of glycerol or the number of freeze–thaw cycles.

#### 2.4.2. Rheological Assay

**Figure 13:**
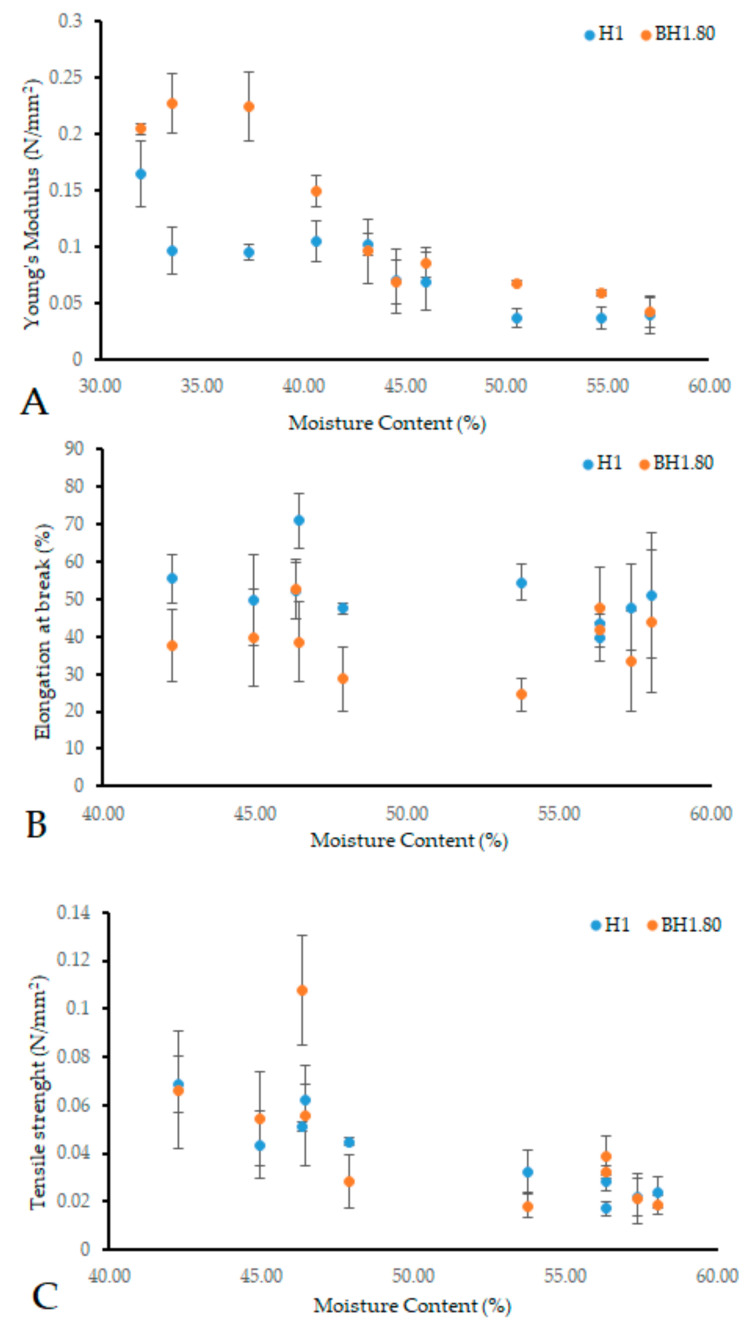
Mechanical properties of sample H1 compared to sample BH1.80 depending on moisture content. (**A**): Young’s modulus (N/mm^2^), (**B**): elongation at break (%), (**C**): tensile strength (N/mm^2^). For each formulation at least eight samples were analyzed in triplicate. Results are presented as mean ± S.D.

Mechanical characteristics are crucial in the design of new biomaterials, including wound dressings, as they must conform to body contours, provide mechanical protection, and maintain their inherent properties during their application period. Key parameters including Young’s modulus, elongation at break, and tensile strength are essential in assessing dressing performance and clinical indications. We evaluated the rheological characteristics of samples BH1.80 and H1, identified as potential optimal wound dressing formulations based on previous assays. Our focus was on rheological properties influenced by moisture content, aiming for a versatile material for both dry and exudative wounds. The addition of COL and HA was found to influence the behavior of the hydrogel membranes, aligning with findings from previous tests. Our analysis revealed that within a moisture content range of 30–40%, the Young’s modulus of sample BH1.80 exceeded that of H1 (Figure 13A). Above a 45% moisture content, this difference diminished, yet BH1.80 consistently maintained a higher modulus of elasticity. As moisture content increased, the elastic modulus declined for both samples, a predictable outcome given that hydrogels soften and gain flexibility upon water absorption. Young’s modulus of healthy human skin typically lies between 0.01–140 MPa, with variations depending on factors such as body location, the orientation of Langer’s lines, age, individual distinctions, and the specific testing method used [153,154,155,156,157,158,159,160]. Elongation at break (Figure 13B) showed that sample H1 had a better performance but again our results revealed that this behavior is dependent on the moisture levels of the samples. Between 40–55%, the difference between the samples was more evident. A moisture content over 55% lead to a reduction in the difference of the elongation at break for the two samples. The elongation capacity of human skin can reach up to 80% of its initial length, while it exhibits a breaking stress of 15 Mpa [161,162]. Our results fall within a desirable range of values of biomaterials that can be used as wound dressings [79,163]. Tensile strength (Figure 13C) for sample BH1.80 exceeded that of sample H1 at moisture levels below 45%. When moisture content ranged from 45–55%, H1 manifested superior tensile strength. Beyond 55% moisture, the difference in tensile strength between the two samples became minimal. The tensile strength values for human skin reported in the literature lie within a broad range [162,163,164]. The observed tensile strength in our study was below the expected level [85,165,166,167,168,169]. However, it is worth mentioning that our primary goal was not to design a product with exceptional mechanical strength. In order to address this finding, we suggest that the obtained hydrogel could be placed on an additional support, usually made of polyurethane [70] if the intended treatment area is subjected to high mechanical stress (such as in the sacral area). In the context of wound dressing applications, our findings indicate that the obtained hydrogel membranes are soft and flexible. These features hold promise for providing increased patient comfort during both application and removal. Furthermore, these hydrogels demonstrate the ability to adapt to a wide range of humidity levels. Additionally, their mechanical properties align with those commonly found in human skin, although variations may occur depending on moisture content and composition.

## 3. Conclusions

The objective of this study was to produce and to explore the influence of polymer ratio and the addition of bioactive agents on the properties of biohybrid hydrogel membranes that can be used as wound dressings. Through a comprehensive analysis using various in vitro and instrumental methods, the study revealed important insights into the performance of these membranes. The hybrid hydrogel membranes displayed cumulative properties, including improved stability and an enhanced swelling ratio. The PVA/Alg ratio had a notable influence especially on the swelling ratio and gel fraction.

The addition of bioactive substances, specifically collagen (COL) and hyaluronic acid (HA), had a significant influence on the performance of the biohybrid hydrogel membranes compared to the hybrid hydrogel membranes. The presence of COL and HA had an impact on the crosslinking process and influenced various characteristics of the obtained membranes, including moisture content, gel fraction, swelling rate, morphological appearance, and mechanical properties.

Interestingly, variations in hyaluronic acid concentrations did not significantly affect the overall properties of the biohybrid hydrogel membranes. 

The introduction of bioactive agents had varying effects on LDH release during cell studies. Cells treated with BH2 formulations (a higher PVA concentration) demonstrated lower LDH release, indicating a decrease in cytotoxicity. On the other hand, cells treated with BH1 formulations (a higher alginate concentration) showed an increase in LDH release. Further research is required to comprehensively understand the underlying mechanisms and fine-tune the concentrations of bioactive agents to enhance biocompatibility.

## 4. Materials and Methods

### 4.1. Materials

Polyvinyl alcohol (molecular weight, 89–98,000; 99% hydrolyzed), bovine serum albumin (BSA, fraction V, 96%), collagenase (Clostridium histolyticum, type IA, ≥125 CDU/mg), CaCl_2_, and hyaluronidase (1000 U) were purchased from Sigma. Sodium alginate, glycerol (anhydrous, 99.0–101%), sodium hyaluronate (≥98.0%), and collagen (rat tail) were purchased from VWR Chemicals, Honeywell, MedChemExpress and Roche, respectively. 

### 4.2. Hydrogel Membrane Production

The biopolymer hydrogel membranes (BP40 and BP50) were prepared via ionic crosslinking, using the solvent casting method. Briefly, the proper amounts (Table 2) from stock solutions (sodium alginate 5% and CaCl_2_ 1%) were dispersed in glycerol. Warm distilled water (68 °C) was added to the homogeneous mixture, and the ensuing solution was homogenized at 1000–1500 rpm for 15 min. The solution was poured onto a Petri dish and left at 28 °C for 48 h. The obtained hydrogel membranes were sterilized by UV light for 15 min and kept at room temperature until further investigations.

The synthetic polymer hydrogel membranes (SP25 and SP15) were prepared using the freezing–thawing (F–T) method reported by Peppas and Stauffer, 1991. The proper amounts of hot PVA solution (10%) and glycerol (Table 2) were mixed with distilled water and homogenized by stirring on a hot plate at 400–600 rpm, for 10–15 min. The solution was poured onto a Petri dish, cooled down at room temperature, and placed at −20 °C, for 20 h. Three F–T cycles (2 h/cycle) were applied to the samples. The hydrogel membranes were sterilized by UV light for 15 min and kept at room temperature.

The hybrid hydrogel membranes (H1, H2, and H3) were prepared by stirring mixtures (according to Table 2) of sodium alginate solution (5%) with PVA solution (10%) prepared in distilled water as described above, at 1500–1800 rpm until the solution became homogeneous. An optimal amount of solution (aprox. 15 g) was transferred onto a Petri dish with a diameter of 5 cm ensuring the uniformity and leveling of the solution. The solution was dried at 28 °C for 48 h, and then it was placed at −20 °C for 20 h. Subsequently, 3 F–T cycles were carried out (2 h/cycle) to ensure crosslinking. The hydrogel membranes were sterilized by UV light, for 15 min and kept at room temperature until further investigations. For the production of the biohybrid hydrogel membranes (BH1.20, BH1.40, BH1.80, BH2.20, BH2.40, and BH2.80) enriched with bioactive compounds COL and HA the polymer solutions were obtained as described above, with the mention that in the final solution the required amounts of COL and HA stock solutions were added, as described in Table 2. The solution was stirred until complete homogenization. The obtained hydrogel membranes were sterilized by UV light, for 15 min and kept at 4 °C until further investigations.

Stock solutions of collagen (0.35%) and hyaluronic acid (0.25%) were prepared in distilled water. 

### 4.3. In Vitro Evaluation of Hydrogel Membranes

#### 4.3.1. Macroscopic Evaluation and pH

The thickness variations were achieved using a digital micrometer (Mitutoyo, Japan) and were evaluated as a dependent variable to optimize the hydrogel formulation. We measured five different points (center and edges). For the weight variation test, the hydrogels were weighed individually. The pH of the hydrogel formulation was determined using a digital pH meter (Thermo Fisher Orion Star A211).

#### 4.3.2. Moisture Content and Moisture Uptake

To determine the moisture content, at the end of the crosslinking process, the samples were cut in the form of a circle with a diameter of 2 cm and weighed before (Wi) and after drying (Wd) [98]. The samples were dried for 24–48 h at 55 °C, until their weight was constant. The moisture content was determined using the following formula:

Moisture Content (%) = (Wi − Wd)/Wi ×100(1)

For moisture uptake determination, the crosslinked hydrogel membranes were cut into circles with a diameter of 2 cm and left for 24–48 h in an oven at 55 °C, until their weight became constant (W1) [96]. The samples were then placed in a chamber with a relative humidity of 75% at a temperature of 24 °C for 72 h. Once the allotted time expired, the samples were carefully removed, wiped of excess water with filter paper and weighed (W2). The moisture uptake ability of the hydrogel membranes was determined using the formula described below:

Moisture Uptake (%) = (W2 − W1)/W1(2)

#### 4.3.3. Swelling Ratio 

The swelling ratio was evaluated in vitro via the gravimetric method [24,170] using simulated wound fluid (SWF) at pH 8.3, and at 37 °C, in an orbital incubator (50 rpm). The simulated wound fluid was prepared in accordance with the method of Arafa et al. [171]. The hydrogel membranes were cut into 2 cm diameter circles and then dried for 48 h at 55 °C until their weight became constant (Wi). The samples were immersed in excess SWF and at predetermined time intervals (5, 30 min and 1, 2, 3, 6, 24, and 48 h) they were removed and weighed (Wf). The excess liquid on the swollen samples was gently removed with filter paper before each determination. The consumed liquid was periodically replaced to ensure the complete immersion of the samples during the course of the test. The swelling rate was calculated using the following formula:

Swelling ratio (%) = (Wf − Wi)/Wi × 100(3)

#### 4.3.4. Biodegradation Study

The biodegradation of the hydrogel membranes was studied in vitro using different solutions—dH_2_O (pH 7.4), SWF (pH 8.3), hyaluronidase solution (HAasa, 10 U/mL), collagenase solution (COLasa, 10 U/mL) and a mixed solution of HAasa and COLasa (HAasa + COLasa, 10 U/mL each)—using an adapted protocol previously described in the literature [172,173]. The enzyme solutions were prepared in simulated wound fluid. The hydrogel membranes were cut into 2 cm diameter circles and then dried for 24–48 h at 55 °C, until their weight became constant (W0). Afterwards, the samples were immersed in the excess of the prepared solutions for 4 h. Once the time had elapsed, the samples were removed from the liquid and the excess was carefully removed with filter paper. Then, they were dried for 24 h at 55 °C and reweighed (Wf). The same samples were then immersed again in the excess of fresh solutions for 20 h, repeating the protocol. The biodegradation rate was calculated using the following formula:

Degradation Rate (%) = (W0 − Wf)/W0 × 100(4)

#### 4.3.5. Gel Fraction

The gel fraction of the hydrogel membranes was evaluated in vitro via the gravimetric method [82,174] using distilled water at room temperature. The hydrogel membranes were cut into 2 cm diameter circles and then dried for 48 h at 55 °C until their weight became constant (Wi). The samples were then immersed in excess distilled water for 24 h and after that they were dried again for 24 h at 55 °C and the final weight was recorded (Wf). The gel fraction was calculated using the following formula:

Gel Fraction (%) = (Wf − Wi) × 100(5)

#### 4.3.6. Water Vapor Transmission Rate

The water vapor transmission rate of the biohybrid hydrogel membranes was assessed using a modified method described by Razzak et al. [175]. To measure WVTR, an Eppendorf tube containing 2 mL of distilled water was utilized, with the hydrogel sample serving as a lid, sealed around the edges. The tube had a diameter of 10 mm, while the sample’s approximate diameter was 12 mm. The weight of each tube was recorded initially (Wi). The tubes were placed in an oven set at 35 °C with a fan speed of 60%. If condensation formed on the membrane surface, it was removed using filter paper. At designated time intervals of 24 and 48 h, the tubes were taken out from the oven and their weights (Wf) were measured. The control was established using Whatman filter paper no. 12. The WVTR was calculated using the following formula:

Water Vapor Transmission Rate = [(Wi − Wt)/A × t] × 10^6^ g/m^2^ h (6)

where A = area of tube opening, and t = time.

#### 4.3.7. Protein Adsorbtion Study

The evaluation of protein adsorption of the hydrogel membranes was assayed using BSA as the protein model. The evaluation protocol employed in this study was adapted from the existing literature [79,127]. The protein was dissolved (5% *w/v*) in phosphate-buffered solutions (PBS, pH 7). Batch experiments were carried out by adding 5 mL of the protein solutions to the hydrogel samples (which had a diameter of 1 cm and thickness of 1 mm). The samples were placed in an orbital shaker (200 rpm) at 37 °C, for 24 h. Aliquots of the solution were collected and initial and equilibrium protein concentrations were assayed spectrophotometrically in a UH5300 Hitachi spectrophotometer (Japan) using the Bradford reagent. The amount of protein adsorbed by the hydrogel membranes was calculated using the following equation:

q = (C_0_ − C_e_) × V/m (7)

where q (mg/g) is adsorption capacity; C_0_ and C_e_ (mg/L) are the initial and equilibrium concentrations of protein in the solution, respectively; V (L) is the solution volume; and m (g) is the hydrogel membrane mass. 

#### 4.3.8. Protein Denaturation Assay

To assess the anti-inflammatory properties of the hydrogels, we evaluated their ability to inhibit protein denaturation. The methodology was adapted from the literature [134,135,136]. The hydrogel samples (with a diameter of 1 cm and thickness of 1 mm) were incubated in a 5 mL solution of 5% BSA in PBS (pH = 7.4) at 200 rpm, and at 37 °C, for 15 min. A solution of 0.5 mg/mL aspirin in PBS was used as a positive control and the negative control was made of BSA solution only. Following the incubation, the samples were subjected to heat treatment at 70 °C for 5 min and then cooled down in an ice bath to reach a temperature of 25 °C. The hydrogel samples were removed and the solution of BSA was evaluated at 278 nm, using a Hitachi spectrophotometer (Japan). For calculating the ability for protein denaturation inhibition, the following formula was used:

Inhibition of denaturation (%) = (1 − At)/Ac × 100(8)

where At represents the absorbance of tested samples and Ac represents the absorbance of the control sample, at 278 nm. 

### 4.4. LDH Assay

For the LDH assay [176], human normal fibroblasts (HS27—CRL-1634-ATCC) were routinely grown in DMEM/F12, supplemented with 10% fetal bovine serum (Corning) in a humidified atmosphere with 5% CO_2_ at 37 °C. Cytotoxicity was assessed via cellular LDH release (CytoTox 96^®^ Non-Radioactive Cytotoxicity Assay, Promega). In total, 10,000 cells were seeded in triplicates overnight in 96-well plates and incubated the next day with triplicates of the gel formulations. Two controls were included—a negative control (cell culture medium only) and a positive control (addition of Cell Lysis Reagent 25x, G182B, Promega, 30 min prior to LDH detection). Additional cell-free wells were incubated with tested gels, for background subtraction. For the LDH analysis, 50 µL of the supernatant was collected from each well and incubated with the assay reagent for 30 min, in the dark. After the addition of the stop solution, absorbance was read at 490 nm with the microplate reader Anthos Zenyth 3100. Cytotoxicity was assessed as a % of that of the lysis control using the described formula:

Cytotoxicity = 100 × (Sample OD − Background OD)/(Average lysis control − Control background)(9)

### 4.5. Instrumental Assay

#### 4.5.1. Morphological Evaluation

The morphological features of the biohybrid hydrogel membranes compared to those of the hybrid hydrogel membranes were acquired using Nova NanoSEM 630 Scanning Electron Microscope (FEI Company, Hillsboro, OR, USA) with an accelerating voltage of 5 kx. For the SEM images, all samples were coat-sputtered with Au to ensure the conductivity of the sample (60 s).

#### 4.5.2. Rheological Assay

The samples were cut into strips (0.5 cm × 4 cm) to analyze the mechanical properties of obtained hydrogels using a protocol adapted from the literature [177,178]. The analyses were performed using the universal testing machine Shimadzu EZ-test SX (100 N) (Kyoto, Japan). Hydrogels were placed between the clamps with an initial separation of 25 mm, and the cross-head speed was set at 10 mm/min. Young’s modulus, tensile strength, and elongation at break were calculated and discussed based on the obtained results.

## Figures and Tables

**Table 1:** Macroscopic evaluation and pH values of hydrogel membranes with different ratios of polymers, with and without bioactive compounds.

Formulation	Weight Variation	Thickness Variation	pH	Homogeneity	Surface	Transparency	Smell	Skin Adhesion/Cooling Effect
BP40	2.55 ± 0.20	1.72 ± 0.04	7.55 ± 0.07	Homogenous	Smooth	Transparent	Specific	Yes
BP50	2.44 ± 0.22	1.92 ± 0.04	7.55 ± 0.07	Homogenous	Smooth	Transparent	Specific	Yes
SP25	0.90 ± 0.02	0.82 ± 0.04	6.23 ± 0.04	Homogenous	Crystalline structures	Transparent	No smell	Yes
SP15	0.88 ± 0.14	0.18 ± 0.04	6.25 ± 0.05	Homogenous	Crystalline structures	Transparent	No smell	Yes
H1	1.31 ± 0.14	1.08 ± 0.11	7.38 ± 0.14	Homogenous	Smooth	Translucent	Specific	Yes
H2	2.12 ± 0.19	1.62 ± 0.16	7.00 ± 0.01	Homogenous	Smooth	Translucent	Specific	Yes
H3	1.92 ± 0.28	1.38 ± 0.11	7.37 ± 0.15	Homogenous	Smooth	Translucent	Specific	Yes
BH1.20	4.73 ± 0.63	1.98 ± 0.08	7.26 ± 0.04	Homogenous	Smooth	Translucent	Specific	Yes
BH1.40	5.69 ± 0.49	2.18 ± 0.15	7.28 ± 0.02	Homogenous	Smooth	Translucent	Specific	Yes
BH1.80	6.21 ± 0.63	2 ± 0.1	7.26 ± 0.04	Homogenous	Smooth	Translucent	Specific	Yes
BH2.20	5.51 ± 0.56	1.84 ± 0.11	7.16 ± 0.03	Homogenous	Smooth	Translucent	Specific	Yes
BH2.40	5.45 ± 0.67	2.24 ± 0.11	7.1 ± 0.01	Homogenous	Smooth	Translucent	Specific	Yes
BH2.80	4.94 ± 0.47	2.1 ± 0.07	7.13 ± 0.01	Homogenous	Smooth	Translucent	Specific	Yes

BP—biopolymer hydrogel membrane, SP—synthetic polymer hydrogel membrane, H—hybrid hydrogel membrane, BH—biohybrid hydrogel membrane. Results are presented as average of triplicates ± S.D.

**Table 2:** Formulation of hydrogel membranes.

Formulation	PVA (mL)	Alginate (mL)	CaCl_2_	Glycerol (mL)	Collagen (mL)	Hyaluronic Acid (mL)
BP40	-	40	10	15.87	-	-
BP50	-	50	10	15.87	-	-
SP25	25	-	-	3.96	-	-
SP15	15	-	-	3.96	-	-
H1	14.3	22.8	7.5	6.8	-	-
H2	22.8	11.4	7.5	6.8	-	-
H3	5.7	45.6	7.5	6.8	-	-
BH1.20	14.3	22.8	7.5	6.8	1.65	0.020
BH1.40	14.3	22.8	7.5	6.8	1.65	0.040
BH1.80	14.3	22.8	7.5	6.8	1.65	0.080
BH2.20	22.8	11.4	7.5	6.8	1.65	0.020
BH2.40	22.8	11.4	7.5	6.8	1.65	0.040
BH2.80	22.8	11.4	7.5	6.8	1.65	0.080

Stock solutions: PVA (10%), sodium alginate (5%), CaCl_2_ (1%), glycerol 99.9%, density = 1.26 g/cm^3^, collagen (0.35%), and hyaluronic acid (0.25%). All formulations were prepared by mixing the stock solutions in the proper amounts presented in Table 2. Final volume = 100 mL. The “-“ sign is used to indicate the absence of a particular substance or component in a given formulation.

## Data Availability

Not applicable.
